# Adaptive Controller for Dynamic Power and Performance Management in the Virtualized Computing Systems

**DOI:** 10.1371/journal.pone.0057551

**Published:** 2013-02-25

**Authors:** Chengjian Wen, Xiang Long, Yifen Mu

**Affiliations:** 1 Department of Computer Science and Engineering, Beihang University, Beijing, People's Republic of China; 2 Department of Computer Science and Engineering, Beihang University, Beijing, People's Republic of China; 3 Academy of Mathematics and Systems Science, Chinese Academy of Sciences, Beijing, People's Republic of China; National Microelectronics Center, Spain

## Abstract

Power and performance management problem in large scale computing systems like data centers has attracted a lot of interests from both enterprises and academic researchers as power saving has become more and more important in many fields. Because of the multiple objectives, multiple influential factors and hierarchical structure in the system, the problem is indeed complex and hard. In this paper, the problem will be investigated in a virtualized computing system. Specifically, it is formulated as a power optimization problem with some constraints on performance. Then, the adaptive controller based on least-square self-tuning regulator(LS-STR) is designed to track performance in the first step; and the resource solved by the controller is allocated in order to minimize the power consumption as the second step. Some simulations are designed to test the effectiveness of this method and to compare it with some other controllers. The simulation results show that the adaptive controller is generally effective: it is applicable for different performance metrics, for different workloads, and for single and multiple workloads; it can track the performance requirement effectively and save the power consumption significantly.

## Introduction

In the past years, more and more interests have been paid to the power and performance management problem in the computer science. The problem is definitely important especially when we realize how much power have been consumed by the data centers worldwide in one year [1]. In this paper, we will study the problem in the virtualized computing system, as the virtualization technology provides an important approach to save energy consumption for a single machine [2] and data center [3]. Based on the adaptive control theory, we will use the least square self-tuning regulator to track the performance, so as to maintain the power consumption at the lowest level which satisfies the performance demand. And we will show that this method is generally effective for different performance metrics, for different workloads, and for single and multiple workloads via simulation.

Although the problem is important undoubtedly, it is indeed complex and hard. This is because of multiple objectives (performance, power, load balance, etc.), multiple influential factors and the nonlinear relationship between them. Literature from the industrial and academic community tried to solve the problem from different aspects. A review on energy-efficient algorithms can be seen in [4].

From the perspective of modeling method, previous work can be classified into several categories as the following: (1). to optimize one objective given constraint on another one. This kind of work might optimize the performance under a power budget, or track the load balance between virtual machines, see [3] [5]–[6] etc.. For example, [3] tracks the utilization using the model predictive control based on different prediction algorithms. [5] considers the correlation between the load balance, the performance, and power and designs a two-layer control structure: first to control the load balance between the virtual machines to track a same performance level by a multi-input-multi-output control approach; then [5] manipulates the frequency. (2). to optimize a new objective, which might integrate several objectives such as the performance, the power, or the balance between different machines, see [7]–[8] etc.. [7] formulates the control problem as a profit maximization problem by integrating the SLA function, which represents the performance, the power consumption, and switching cost. Then two-level control hierarchy is introduced, where one level is a faster control, the other level is a slower control. [8] defines a cost function which integrates the performance and power consumption.

From the perspective of regulating method, pervious work can be classified into several kinds too: (1). via control theory, like the optimal control, see [3] [5]–[8] etc.. Multiple kinds of controller are designed, such the feedback controller, the optimal controller, the Proportional-Integral-Derivative(PID) controller, the Model-Predictive Controller etc.. For example, [3] compares the results by different controllers and find that the predictive controller performs better with some self-learning behavior. [6] uses the PID controller and model predictive controller at the same time. (2). via the heuristic algorithms, see [9]–[10] etc.. For example, [10] defines a cost function for the long run, which includes both of the reward and penalty in the future, then [10] uses the reinforcement learning method together with fuzzy rule bases to achieve the defined objective. Among these methods, control theory has been applied more and more as it can provide an unified framework and a rigorous controller design. Different from the above engineering aspect, [11]–[12] investigate the abstraction of load balance problem: the balls into bins, and investigate it from a theoretical point based on a probability framework.

In this paper, we will investigate the performance and power problem in the virtualized environment based on adaptive control theory. To be specific, we will formulate the problem to optimize (minimize) the power consumption, and make sure that the performance satisfies a given requirement. That is quite practical in a real system. Then the adaptive controller based on the least-square self-tuning regulator (LS-STR) will be designed for the first time to adaptively and dynamically track the performance. We will show the effectiveness of this method by simulation, in which, the performance model and the power model will be built based on data collected from real machines. The simulation results show that the adaptive LS-STR is generally effective and has obvious advantages to other controllers: it is very general and flexible and is easy to implement; it can track different performance metrics for single or multiple different jobs effectively, which is better than the predictive controller; it can save power consumption considerably compared to the open loop controllers; and finally, it needs only the input and output information while the system model and the workload info is not required, thus it is applicable for a lot of practical problems.

The paper makes special contributions in the following points:

1. By defining the integrated resource for each applications, the problem can be solved in two steps: the performance tracking and power minimization. Then, to track the different performance metrics, we design the adaptive controller based on LS-STR for general systems, which can estimate the system model and track the performance at the same time.

2. In order to test the effectiveness of the adaptive controller, we design a series of simulations. In simulation, there can be single or multiple workloads, the performance metric can be different too. Then the simulation results show that the adaptive controller is generally effective to track the performance and thus save the power.

## Analysis

### The system and resource

**Figure 1:**
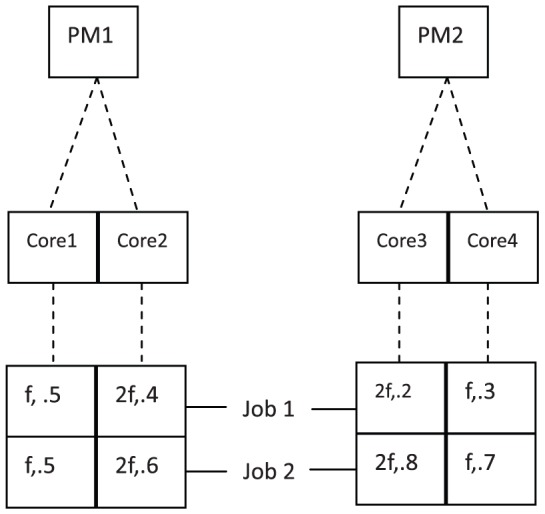
The hierarchical structure of the system. Two physical machines(PM) are in the system with two cores on each PM.

A typical virtulized computing system can be illustrated by Figure 1, from which we can see a clear hierarchical architecture between the resources.

On the top level, there are 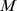 physical machines(PM), denoted as 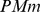. Each PM has certain number of cores. Assume there are 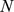 cores all together, denoted each as 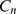.

Each core has its feasible frequencies when it is running, which might be different from each other. Usually, the feasible frequencies form an arithmetic sequence, i.e., the frequency can be 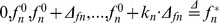. Generally, 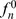, 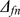, 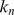, 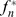 are same for the cores on the same PM, but can be different from cores on different PMs. When the frequency is 0 while the PM is still powering on, the PM is called to be at the idle state, at which the power consumption is positive.

Now the virtual machines can be defined and built on the cores. A virtual machine (VM) is an abstraction of the physical machine, which can be run on a PM and can be migrated between different PMs. Usually, a VM is supposed to carry out a specific kind of service or applications (we call them a job), such as the website request, computing demand and so on. The VM makes it possible to share computing resources on a PM/core among multiple applications and shut down the idle PMs thus can save power consumption.

Suppose 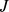 jobs are arriving and waiting for resources. The virtual machine for the *j*th job on the 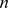
th core can be denoted as 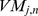, if it exists. To complete the *j*th job on 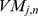, two factors of a core 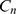 will mainly influence the performance: the frequency of the core 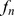, and the CPU share 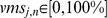. Both of them can be regulated. Obviously, the CPU share satisfy 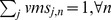.

To summarize briefly, we can define the resource vector 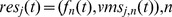 as the resource allocated to the *j*th job on the 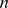
th core. And 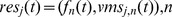 catches the main influential factors to complete the job.

The above definitions can be illustrated by Figure 1 in detail. In Figure 1, two PMs are in the system with two cores on each PM. So the cores are denoted as 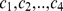. There are two jobs demanding the resource. To complete the first job, the VMs are built on each core and the resource vector for the first job is 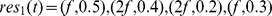.

### Problem statement

In the real problems, the strength of the *j*th job, which is called the workload and denoted as 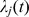, is usually time-varying, stochastic and sometimes is periodic. Thus it is necessary to regulate the resource vector dynamically in order to gain performance and save power.

There are several criteria to represent the performance depending on the property of specific jobs. The most popular criteria in the literature are the response time 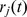 and the throughput 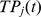, both of which is measurable in real time. The smaller response time implies the fast processing rate, while the bigger throughput implies the bigger processing capability. Both of them imply a good performance.

The power consumption can be represented by the power value 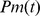
(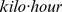) for each physical machine and it can be measured in real time by power meters ([13]).

Generally, a tradeoff exists between high performance and low power consumption: high performance means more energy consumption. In the real problems, we usually place the performance demand prior to the power consumption. For example, usually we require that 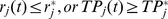. In order to save power as much as possible, we try to make the performance to exactly satisfy the requirement. Hence, the problem can be formulated as
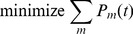
(1)

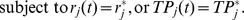



In the real problems, the dynamical regulation of the resource vector 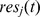 can be carried out from bottom level to the top level of Figure 1: first, to regulate the CPU share; second, to regulate the frequency; third, to turn on/off the VMs; and finally, to turn on/off the physical machine. Apparently, it is not easy to solve such a problem.

## Methods

We will solve the problem (1) by two steps: first, to track the performance; second, to minimize the power consumption. To make this method feasible, we define the integrated resource as
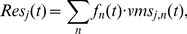
(2)


which is a scalar and will be the bridge between the two steps. In the following we will give the solutions of the two steps. First, we will track the performance.

In many cases of real problems, the workload 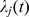 can not be measured because of the time delay or some system constraint. For this case, we can apply the adaptive control theory to track the performance. In this section, we will use 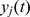 and 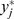 to represent the performance 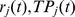 and performance requirement 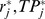.

With the definition of 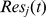, we can build the linear regression model between the resource and the performance as below:
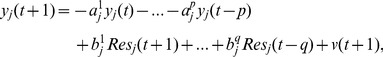
(3)


where 
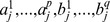
 are coefficients, 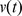 is the noise which covers the stochastic and other effects. Denote 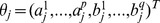 and 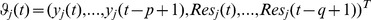, then (3) can be simplified as 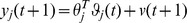.

The linear regression model (3) above is a very general model. It models the relationship between the input 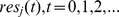 and the output 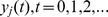, and the relationship can be definitely nonlinear. On the other hand, it is a simple model, which make it easy to design the adaptive tracker for the model.

For the system (3), we can design the least square self-tuning regulator (LS-STR) to track the output 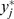:
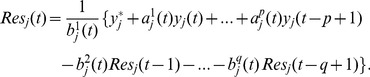
(4)


where 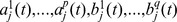 are the estimates of the parameters which are obtained by the iterative least square algorithm as below
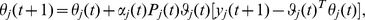


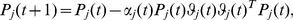
(5)

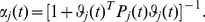



The initial values 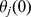,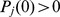 can be taken arbitrarily.

From [14], under some weak and natural conditions on 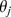, the output 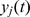 is optimal in the sense that the accumulated close-loop tracking error 
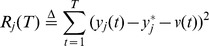
 holds that 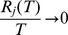 with respect to 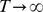.

To make it clear, the idea of self-tuning regulator dates back to 60 years ago([15]) and was discussed much in the following, like [16] etc. Then [14] [17]–[18] proved the global stability and optimality of the LS-STR and describe the convergence rate of it. Since it is very flexible and very easy to implement, it has found the application in many fields, such as the steel rolling, paper making, metallurgy, automatic pilot of mammoth tanker, etc. ([19]). This paper can be regarded as a new application of LS-STR.

So far, by defining the integrated resource 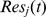 and build the linear regression model (3), the adaptive controller based on LS-STR (4) (5) can be designed which can adaptively track the reference output 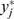 by regulating the integrated resource while estimating the parameters on line at the same time.

In the simulation, for all the jobs, we take 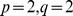 base on several trials.

Now suppose we have gotten 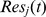 computed by LS-STR (4). Then there might be multiple 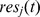 satisfying 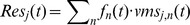 while they result in different power consumptions. Thus we need to find the ‘good’ 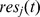 to minimize power consumption. This can be formulated as an optimization problem below:
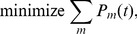
(6)

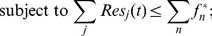
(7)

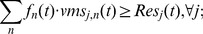


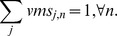



In (6), 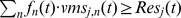 since the performance constraint must be satisfied. The solution to the problem (6) will be taken as the solution to the original problem (1).

## Experiments

### Basic settings

To test the effect of the adaptive strategy to manage the performance and power, we design some simulations with single different workloads, different performance criteria, and multiple workloads.

Suppose in a virtualized computing environment, there are two physical machines, with one core on each machine. For the sake of simplicity, we assume that each core has the same four feasible frequencies: 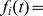 0, 1.6 g, 2.2 g, 2.8 g, 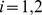. This setting is simple but remains the generality and difficulty of the problem.

### Performance model

The performance model will be used to generate the system in the simulation. Meanwhile, the performance model is necessary to design the predictive controller to track the performance.

In Section 3.2, we have stated that there are two typical performance criteria which are usually used in the literature: the response time and the throughput. Both of them will be adopted in the simulation.

To build the performance model, first note that for a given resource 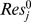, both the response time and the throughput will encounter a critical value 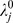, which can be regarded as the maximal capacity corresponding to 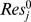.

Then, concerning the response time of a specific job, when the workload is below the maximal capacity, a liner model can be built to approximate the relationship between the performance, the resource and the workload. When the workload is very large, the response time will increase significantly ([7]).

Suppose the maximal capacity of a core/PM with the resource being 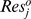 ( = 2.8 here) is 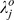, the corresponding response time is 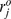 (taken as 3 seconds in simulation). Then, with the workload 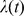 and the resource 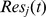, the response time can be roughly modeled as
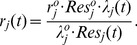
(8)


Concerning the throughput, when the workload is below the maximal capacity, all the workload can be dealt with. When the workload is larger than the maximal capacity, then the workload which is beyond the maximal capacity will be abandoned. Thus, the throughput can be modeled as below:
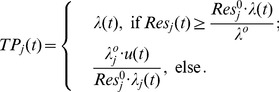
(9)


It is notable that the maximal capacity 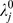 obviously is related to the special PM and the job's type. And in simulation, we assume the workload is always below the maximal capacity.

Additionally, from the performance model (8) the response time is inverse to the resource 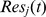, so when the response time is considered, we will take 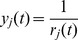 as the performance output in (3) in order to avoid bad tracking effects. Similarly, when the throughput is considered, we will take 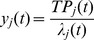 as the performance output in (3).

### Power model

The power model is the base to solve the optimization problem (6). Roughly, the current power of the physical machine, 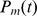, mainly depends on the normalized CPU share of the physical machine 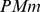 ([20]), which is defined as
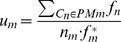
(10)


and includes both the CPU share and the CPU frequency together. Obviously, 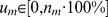, 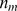 is the number of cores of 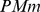.

Then the power for a physical machine can be modeled to be linear with 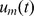:
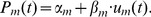
(11)


The coefficients 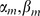 can be obtained by regressing the data of the measured power 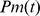 and CPU utilization 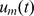.

In the simulation, data are collected from a Dell R510 and the power models are taken as
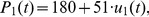
(12)

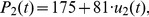
(13)


where 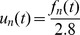, 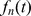 is the frequency of the core. It is easy to see that in a system, the power consumption can vary significantly with a same resource configuration.

### Workloads

In the simulation, we will choose two different types of workloads as the testcases. They are the website request rates from a university [21] for two different traffics. Data are collected every 5 minutes for 2 days, so there are 574 data for each workload.

**Figure 2:**
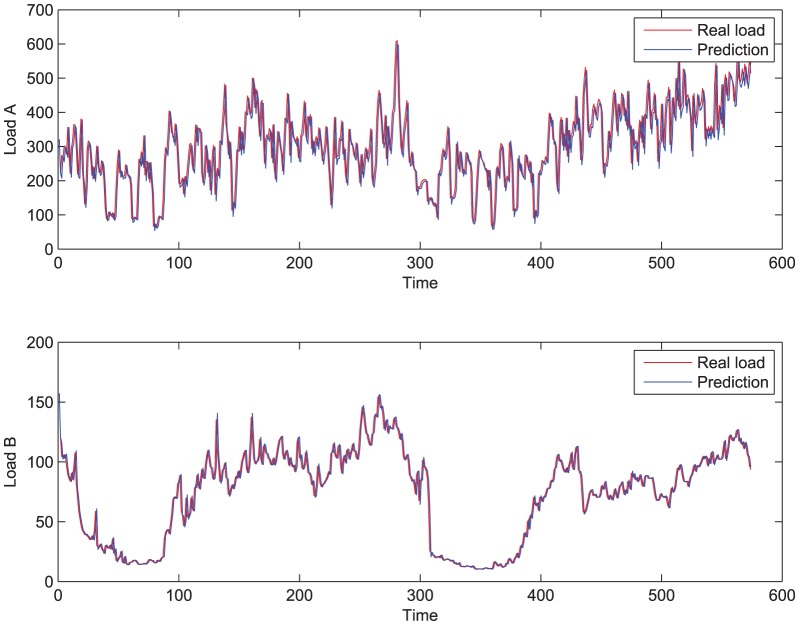
The workloads and predictions with ar(2) model. The workloads are predicted with ar model for one step.

Figure 2 below shows the sequence of the two workloads: load A, load B. And in the following, we just write as 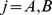 when necessary. Apparently, they are very different: although neither of them is stationary, load A is more stationary than load B; load B is nearly periodic along with the time. In the simulation, we adopt different performance metrics for them: for load A, we consider the throughput; for load B, we consider the response time.

When the workload can be measured, however, that is rarely true in real problems, it can be predicted by suitable model. Then the predictive controller can be designed to track the performance. In the simulation, we will use the AR(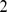) model to predict the workload:
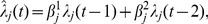
(14)



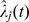
 is the prediction. The coefficients 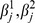 can be estimated by online or off line algorithms, see [22].

For instance, when we use the first 100 statistics to estimate 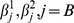, for load B by off-line algorithm, we get 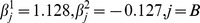. Then, together with the response time model (8), we can figure out the desired resource to ensure the performance requirement:
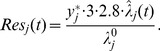
(15)


Here AR(2) model is adopted because it is sufficient to make prediction and it is simple enough.

## Results and Discussion

**Figure 3:**
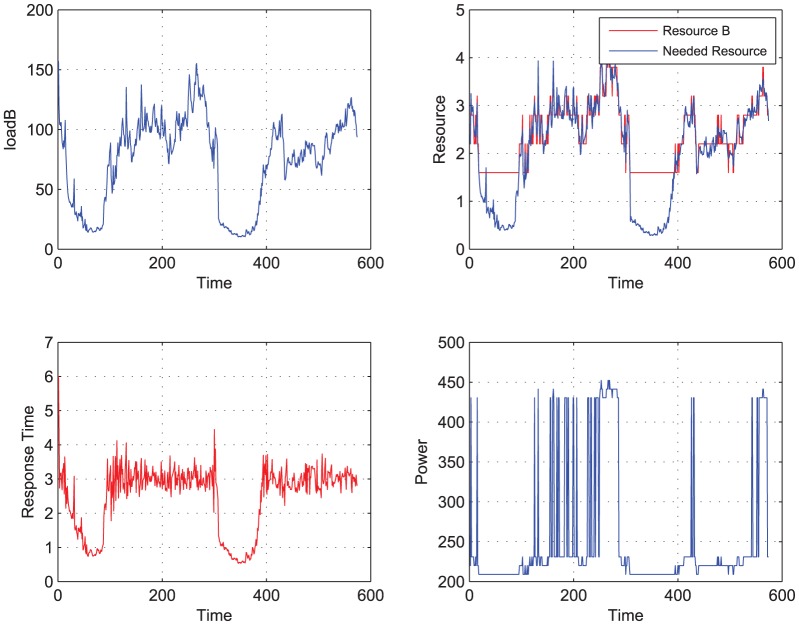
Load B to be completed, using predictive controller. When Load B is performed the power and performance are traced. Meanwhile the ideal needed resource and the actual resource allocated by the controller are both shown in this figure.

**Figure 4:**
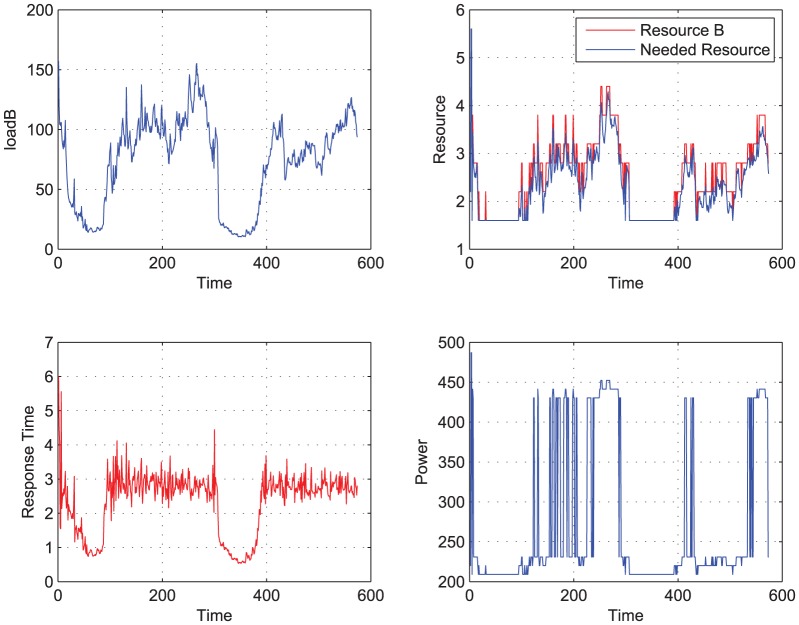
Load B to be completed, using LS-STR. When Load B is performed the power and performance are traced. Meanwhile the ideal needed resource and the actual resource allocated by the LS-STR controller are both shown in this figure.

**Figure 5:**
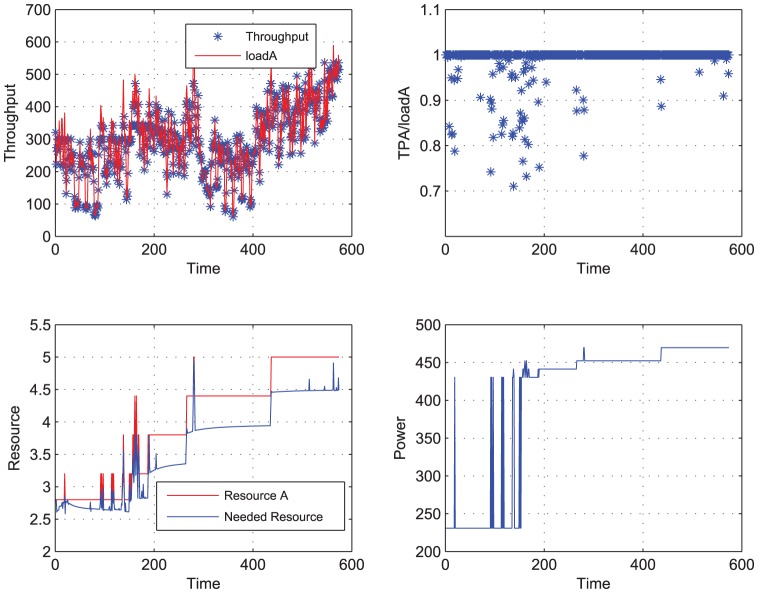
Load A to be completed, using LS-STR. When Load A is performed the power and performance are traced. Meanwhile the ideal needed resource and the actual resource allocated by the LS-STR controller are both shown in this figure.

**Figure 6:**
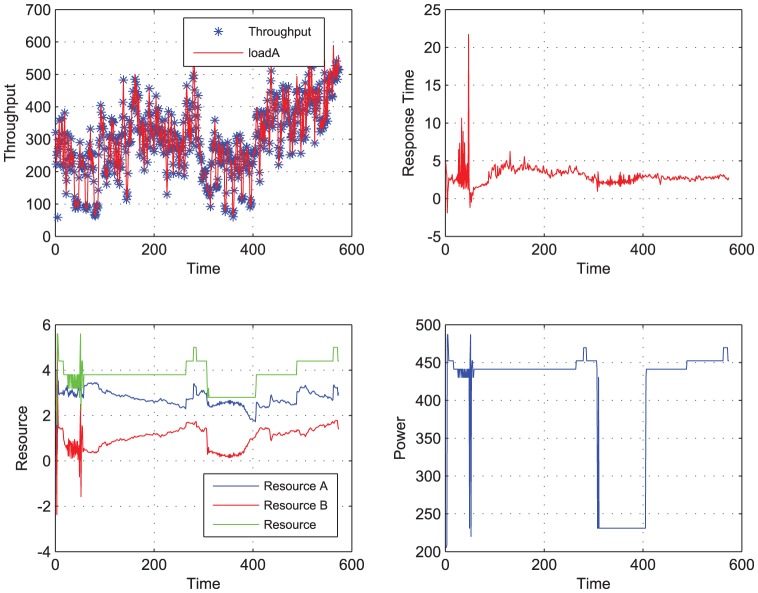
Two loads to be completed using LS-STR. When Load A and load B are performed the power and performance are traced. Meanwhile the ideal needed resource and the actual resource allocated by the LS-STR controller are both shown in this figure.

Figure 3, Figure 4, Figure 5, Figure 6 below show the simulation results. We will illustrate them in the following to show that the adaptive controller based on LS-STR is suitable and effective to track the performance and save power, whatever the cases is when there are one single workload or multiple workloads, or when the different performance metrics are adopted.

### A: single load: load B, to track the response time

Now since there is only one type of workload: load B, we have 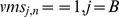. The parameters in (8) are taken as 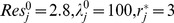.

Now the response time 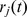 is adopted as the performance metric, and 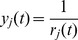 is the performance output in the regression model (3). And the controllers are designed to track the reference output 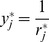.

We will use three types of controllers: the open loop controller, the predictive controller and the adaptive controller based on LS-STR to track the performance. When the open loop controller is used, the core will always be running with the highest frequency, i.e., 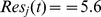. When the predictive controller is used, the resource will be obtained from (15). When the the adaptive controller based on LS-STR is used, the resource is obtained from (4)(5).

Figure 3, Figure 4 shows the simulation results using the predictive controller and adaptive controller. Table 1 lists the main indices of the simulation.

**Table 1:** The performance and power under three controllers; load B, response time tracked.

controller	perf mean	perf variance	perf satisfy	power mean	power save
open-loop	1.1111	0.3095	574	487	0
predictive controller	2.4946	0.8861	369	253.9633	47.85 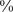
LS-STR	2.3529	0.7725	479	272.3558	44.07 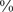

When load B is performed the performance metric is response time. Performance and power comparisons are listed under these three controllers.LS-STR controller has the best power saving result.

From Figure 3, Figure 4, and Table 1, we can see that the adaptive controller based on LS-STR has obvious advantages on managing the performance and power with less information cost compared with the open loop controller and the predictive controller. In details,

(1). Using the open-loop controller, the averaged response time is 1.1111 with the variance being 0.3095. The performance requirement is satisfied at each time. However, the power is the maximal 487 at each time.

(2). Using predictive controller, the averaged response time is 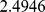, with the variance being 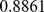. There are 369 times satisfying the demand. The mean power consumption is 253.9633 and is saved by 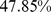 compared to the open loop controller.

Considering that the accurate performance model can not be obtained in the real experiment, the predictive controller is very possible to perform worse.

(3). Using the adaptive controller based on LS-STR, the average response time is 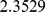 with the variance being 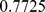, both of them are smaller than that using predictive controller. And in the response time sequences, those which satisfy the demand has a number of 479, which is much larger than using predictive controller. Now the averaged power consumption is 272.3558, and is saved by 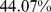 compared to the open loop controller. Note that this reduction is quite considerable since the total power consumption can be very large according to [1].

So, to summarize briefly, LS-STR can achieve a much better performance than the predictive controller. At the same time, LS-STR can save power consumption as big as 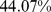 compared to the open loop controller, which is as good as the predictive controller. On the other hand, LS-STR does not need to know the accurate performance model or to measure the workload, which is the situation in many real problems and thus it can be applied to a lot of systems. These show the great advantage of the adaptive controller based on LS-STR to other controllers like open loop controller and predictive controller.

### B: single workload: load A, to track the throughput, using LS-STR

Still, since there is only one type of workload: load A, we have 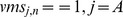. The parameters in (9) are taken as 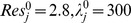.

Now the throughput 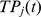 is adopted as the performance metric, and 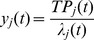 is the performance output in the regression model (3). Now the LS-STR is designed to track the reference output 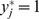.

Figure 5 and Table 2 below show the simulation result using the adaptive controller based on LS-STR.

**Table 2:** The performance and power using LS-STR: load A, to track the throughput.

workload	mean(load A)	mean( 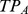 )	mean( 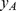 )	var( 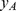 )	perf satisfy	power save
load A	301.3564	296.6247	0.9886	0.0420	509	17.97%

When load A is performed the performance metric is throughput. LS-STR controller shows 17.97% power saving.

From Figure 5 and Table 2, we can see that the adaptive controller based on LS-STR is effective to track the throughput, which is quite different from the response time metric in both definition and the applicable scope. To be specific, when we use the LS-STR to track the throughput of load A with its mean being 301.3564, the throughput sequence 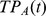 can get a mean being 296.6247, while the performance output 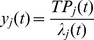 has a mean being 0.9886 with a variance 0.0420, which is very near the required value 1. And along the time, for as many as 509 times, the throughput equals the workload, i.e., no workload is abandoned. And this controller can save power 17.97% compared with the open loop controller.

### C: two workloads, two performance metrics, using LS-STR

Now, since there are two types of workload to be dealt with in the system: load A and load B. So we have 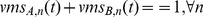. Thus now the 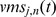 can be taken as the real number in the interval [0,1]. Now the parameters in (9) are taken as 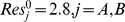, 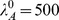, 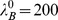, 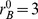.

Now the throughput 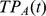 is adopted as the performance metric for load A, and the response time 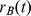 is adopted as the performance metric for load B, and 
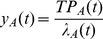
, and 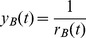 are the respective performance output in the regression model (3). And the reference output is 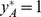 and 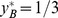.

In the simulation, to solve the optimization problem (6), we first get 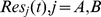, then we get the total resource 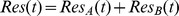. Note that such a 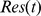 can be out of the feasible set since the frequency set is discrete here. Then we find the 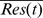 belonging to the feasible resource set which is nearest to and bigger than the required 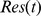. Then the resource 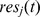 will be allocated to the jobs according to the proportion, i.e. 
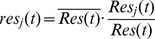
.

Figure 6 and Table 3 below show the simulation result using the adaptive controller based on LS-STR.

**Table 3:** The performance and power using LS-STR: two loads, two performance metrics.

workload	perf mean	perf variance	perf satisfy	power save
load A	mean( 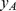 ) = 0.9962	var( 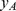 ) = 0.0013	549	16.43 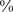
load B	mean( 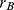 ) = 2.9905	var( 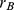 ) = 1.6145	332	16.43 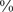

Load A and load B are performed at the same time and LS-STR controller shows 16.43% power saving.

From the Figure 6 and Table 3, we can see that the adaptive controller based on LS-STR is also effective to deal with the situation where there are two workloads in the system and two performance metrics in the system. To be specific,

(1). the LS-STR can track the throughput of load A pretty well: the sequence 
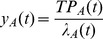
 has an averaged value 0.9962 with a variance 0.0013, and along the time, for as many as 549 times, the throughput equals the workload, i.e, no workload is abandoned.

(2). the LS-STR can track the response time of load B well too: the response time sequence 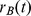 has an averaged value of 2.9905 with a variance 1.6145, and along the time, for as many as 332 times, the response time is smaller than 3 seconds, implying that the performance requirement is satisfied.

(3). And finally, the LS-STR can save 16.43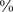 power consumption compared with the open loop controller.

Of course, the curve of the changing 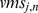 can also be drawn to see the details of the simulation process, which is omitted here.

## Conclusions

Performance and power management in the virtulized environment is a fundamental, important and difficult problem. In this paper, by designing the adaptive controller based on least square self-tuning regulator (LS-STR), we can dynamically regulate the resources and thus track the required performance and keep the power at a lower level as desired. Simulation results show that this method is very effective and general: it can deal with the problem when there are one application or multiple applications; it is also effective for different performance metrics.

Much work are worthy to do to complete the result and improve the solution in the future. For example, we can study how the parameters influence the effect of LS-STR. And when the maximal capacity of the system is not enough for the applications, there exists conflicts and games between the applications, so the applications might struggle for the resource. We can also take the time-delay effect and the switching cost when we turn on/off the PM into account. Moreover, in the real systems, both the performance and power models can be different among the physical machines and the topological structure between the physical machines also influence the performance and power. All these things will make the problem challenging in theory as well as engineering.

## References

[1] Growth in data center electricity use 2005 to 2010. Available: http://www.analyticspress.com/datacenters.html. Accessed 2012 May 1.

[2] Wen C, He J, Zhang J, Long X (2010) PCFS: Power Credit Based Fair Scheduler Under DVFS for Muliticore Virtualization Platform. Proceedings of the 2010 IEEE/ACM Int'l Conference on Green Computing and Communications & Int'l Conference on Cyber, Physical and Social Computing 163–170.

[3] Xu W, Zhu X, Singhal S, Wang Z (2006) Predictive Control for Dynamic Resource Allocation in Enterprise Data Centers. Proceedings of the 10th IEEE/IFIP Network Operations and Management Symposium 115–126.

[4] Albers S (2010) Energy-efficient algorithms. Communications of the ACM 53(5): 86–96.

[5] Wang Y, Wang X, Chen M, Zhu X (2008) Power-Efficient Response Time Guarantees for Virtualized Enterprise Servers. Proceedings of the 2008 Real-Time Systems Symposium 303–312.

[6] Lim H, Kansal A, Liu J (2011) Power Budgeting for Virtualized Data Centers. Proceedings of the 2011 USENIX conference on USENIX annual technical conference 5–15.

[7] Kusic D, Kephart JO, Hanson JE, Kandasamy N, Jiang G (2009) Power and Performance Management of Virtualized Computing Environments Via Lookahead Control. Journal Cluster Computing 12(1): 1–15.

[8] Wang X, Chen M (2008) Cluster-level feedback power control for performance optimization. Proceedings of the 14th IEEE International Symposium on High Performance Computer Architecture 101–110.

[9] Xu J, Fortes JAB (2010) Multi-Objective Virtual Machine Placement in Virtualized Data Center Environments. Proceedings of the 2010 IEEE/ACM Int'l Conference on Green Computing and Communications & Int'l Conference on Cyber, Physical and Social Computing 179–188.

[10] Vengerov D (2007) A reinforcement learning approach to dynamic resource allocation. Journal Engineering Applications of Artificial Intelligence 20(3): 383–390.

[11] Bender MA, Rabin MO (2000) Scheduling cilk multithreaded parallel programs on procesors of different speeds. Proceedings of the twelfth annual ACM symposium on Parallel algorithms and architectures 13–21.

[12] Wieder U (2007) Balanced allocation with heterogeneous bins. Proceedings of the nineteenth annual ACM symposium on Parallel algorithms and architectures 188–193.

[13] Krishman B, Amur H, Gavrilovska A, Schwan K (2010) VM power metering: feasibility and challenges. ACM SIGMETRICS Performance Evaluation Review archive 38(3): 56–60.

[14] Guo L (1995) Convergence and logarithm laws of self-tuning regulators. Automatica 31(3): 435–350.

[15] Kalman RE (1958) Design of a self-optimizing control system. Trans. ASME 80: 468–478.

[16] Astrom KJ, Wittenmark B (1973) On self-tuning regulators. Automatica 9: 185–199.

[17] Guo L, Chen H (1991) The Astrom-Wittenmark self-tuning regulator revisited and ELS-based adaptive trakers. IEEE Trans. Automatic Control 36(7): 802–812.

[18] Guo L (1994) Further results on least square based adaptive minimum variance control. SIAM J. Control and Optimization 32(1): 187–212.

[19] Astrom KJ (1983) Theory and appliction of adaptive control-a survey. Automatica 19: 471–486.

[20] Spec website. Available: http://www.spec.org/power_ssj2008/results/res2010q4/power_ssj2008-20100921-00294.html. Accessed 2012 Jun 6.

[21] Beihang University Website.Available: http://mrtg.buaa.edu.cn.Accessed 2012 Jun 10.

[22] Ljung L (1999) System identification: Theory for the User. N.J: Prentice Hall. 20 p.

